# PREVALENCE OF DEMENTIA AND COGNITIVE IMPAIRMENT IN PUBLIC SENIOR HOUSINGS: A SYSTEMATIC REVIEW AND META-ANALYSIS

**DOI:** 10.1093/geroni/igad104.2830

**Published:** 2023-12-21

**Authors:** Soobin Park, Takashi Amano, Sojung Park, BoRin Kim

**Affiliations:** Washington University in St. Louis, St. Louis, Missouri, United States; Rutgers University, Newark, New Jersey, United States; Washington University in Saint Louis, St. Louis, Missouri, United States; University of New Hampshire, Durham, New Hampshire, United States

## Abstract

Older residents in public housing represent a particularly vulnerable population at risk for cognitive decline due to their disadvantaged socioeconomic and health-related characteristics which are well-known risk factors of dementia. This study aims to synthesize available evidence regarding the prevalence of dementia and cognitive impairment among public senior housing residents. We conducted a literature search for studies published in English in the following databases: PsychInfo, PubMed, MEDLINE, CINAHL, and The Cochrane Library. We focused on observational studies about residents of public senior housings that reported prevalence and/or incidence of dementia and/or cognitive impairment screened by assessment tools for detecting dementia equivalent cognitive impairment. A total of ten studies were included for a meta-analysis to estimate the overall prevalence of cognitive impairment and dementia. Seven of ten studies utilized screening tools to detect dementia equivalent cognitive impairment. Overall pooled prevalence of cognitive impairment or dementia was 20.4% (95%CI: 14.1-27.5). The subgroup analyses revealed the pooled prevalence of cognitive impairment among residents of public senior housing was 25.5% (95%CI: 22.0-29.1) and pooled prevalence of dementia was 13.5% (95%CI: 8.3-19.7). The subgroup findings suggest the prevalence of dementia may be higher among residents of public senior housing compared to their peers in non-public housing. Also, this population may be particularly susceptible to an unrecognized or delayed diagnosis of dementia due to the lack of formal detection strategies in public senior housing. More resources should be allocated for detection to ensure better support the residents.

